# Coxsackievirus B3 Responds to Polyamine Depletion via Enhancement of 2A and 3C Protease Activity

**DOI:** 10.3390/v11050403

**Published:** 2019-04-30

**Authors:** Courtney N. Dial, Patrick M. Tate, Thomas M. Kicmal, Bryan C. Mounce

**Affiliations:** 1 Department of Microbiology and Immunology, Stritch School of Medicine, Loyola University Chicago, Maywood, IL 60153, USA; cdial@luc.edu (C.N.D.); ptate@luc.edu (P.M.T.); tkicmal@luc.edu (T.M.K.); 2 Infectious Disease and Immunology Research Institute, Stritch School of Medicine, Loyola University Chicago, Maywood, IL 60153, USA

**Keywords:** polyamines, Coxsackievirus B3, protease

## Abstract

Polyamines are small positively-charged molecules abundant in eukaryotic cells that are crucial to RNA virus replication. In eukaryotic cells, polyamines facilitate processes such as transcription, translation, and DNA replication, and viruses similarly rely on polyamines to facilitate transcription and translation. Whether polyamines function at additional stages in viral replication remains poorly understood. Picornaviruses, including Coxsackievirus B3 (CVB3), are sensitive to polyamine depletion both in vitro and in vivo; however, precisely how polyamine function in picornavirus infection has not been described. Here, we describe CVB3 mutants that arise with passage in polyamine-depleted conditions. We observe mutations in the 2A and 3C proteases, and we find that these mutant proteases confer resistance to polyamine depletion. Using a split luciferase reporter system to measure protease activity, we determined that polyamines facilitate viral protease activity. We further observe that the 2A and 3C protease mutations enhance reporter protease activity in polyamine-depleted conditions. Finally, we find that these mutations promote cleavage of cellular eIF4G during infection of polyamine-depleted cells. In sum, our results suggest that polyamines are crucial to protease function during picornavirus infection. Further, these data highlight viral proteases as potential antiviral targets and highlight how CVB3 may overcome polyamine-depleting antiviral therapies.

## 1. Introduction

Coxsackievirus B3 (CVB3) is a positive-sense RNA virus, belonging to the *Picornaviridae* family. CVB3 commonly infects children, resulting in a self-limiting illness that can lead to the development of muscle, lung, and heart maladies. Importantly, CVB3 infection can lead to myocarditis and endocarditis, and the virus is able to persist in cardiac tissue [1,2]. Currently, no antivirals are available for treating or preventing CVB3 infection; thus, the identification of novel antiviral targets is crucial to controlling infection.

Viral proteases, including enteroviral proteases, play crucial roles in the replication of RNA viruses [3]. Following virus entry, picornavirus RNA is directly translated via host ribosomes into a polyprotein, containing both structural and nonstructural proteins. This large protein is subsequently cleaved to generate functionally distinct viral proteins. Importantly, the ability of viral proteases to cleave the viral polyprotein is essential for productive infection, as limiting viral protease activity significantly hinders virus infection. Modulation of protease activity, however, can also alter antiviral susceptibility [4]. Additionally, viral proteins cleave several host proteins, including translation factors, immune effectors, and signaling molecules [5,6,7,8,9,10], to establish a proviral environment.

Viral proteases play many roles during infection, and picornaviruses encode two distinct proteases: 2A (2A^pro^) and 3C (3C^pro^). The 2A and 3C picornaviral proteases function to cleave both host and viral proteins. 3C is responsible for the majority of viral polyprotein cleavage. Importantly, 3C also functions as part of 3CD (a polyprotein consisting of the 3C protease and 3D polymerase) and acts as a protease in this context as well [11]. To date, reports demonstrated that 2A facilitates the cleavage between the P1 and P2 protein segments [12]. The precise regulation of these proteases, their activities, and their overlapping and unique functions remains to be fully understood. However, due to their copious functions during infection, they represent potential drug targets [13,14,15].

Polyamines are small, positively-charged molecules that are abundant in eukaryotic cells. Polyamines function in nucleotide metabolism, cell cycling, and cell signaling, among several other functions [16,17,18]. Several compounds have been developed that specifically target polyamine metabolism, either by reducing their synthesis or enhancing their breakdown, and these compounds have been tested clinically for the treatment of cancers and parasites [19,20,21]. Difluoromethylornithine (DFMO), which inhibits polyamine biosynthesis, has received attention as an anti-trypanosomal molecule and is well-tolerated in patients [22,23]. Polyamines are also crucial for RNA virus infection, including CVB3 [24]. Polyamine depletion via DFMO restricts CVB3 infection, in vitro and in vivo [25]. We previously described how Chikungunya and Zika viruses rely on polyamines for viral translation and genome replication [26]; however, whether similar processes are affected for other viruses and precisely how polyamines function during CVB3 replication remain to be completely understood.

To investigate how polyamines facilitate CVB3 infection, we performed a screen for virus escape mutants that were resistant to DFMO treatment. We isolated two distinct viral mutants in the 2A and 3C proteases. We determined that these mutant viruses were resistant to polyamine depletion and that these mutations conferred no fitness advantage in the absence of polyamine depletion. To investigate the proteolytic activity of these enzymes, we developed a protease-dependent split-luciferase reporter system to measure 2A and 3C activity. Using this assay, we observed that 2A and 3C activity was diminished with polyamine depletion, to which mutant 2A and 3C proteases were resistant. Additionally, we observed that viruses containing these mutants were able to cleave cellular targets efficiently in polyamine-depleted cells. Together, these data suggest that polyamines are crucial for viral protease activity and that we may target CVB3 proteolytic activity through polyamine-depleting pharmaceuticals like DFMO.

## 2. Materials and Methods

### 2.1. Cell Culture

Cells were maintained at 37 °C in 5% CO_2_, in Dulbecco’s modified Eagle’s medium (DMEM; Life Technologies, Waltham, MA, USA) with bovine serum and penicillin-streptomycin. Vero cells (BEI Resources, Manassas, VA, USA) were supplemented with 10% new-born calf serum (NBCS; Thermo-Fisher, Waltham, MA, USA). HeLa and 293T cells, kindly provided by Dr. Ed Campbell, were supplemented with 10% fetal bovine serum (FBS; Thermo-Fischer).

### 2.2. Generation of CVB3 and 2A^29K^ and 3C^52R^ Mutants

CVB3 (Nancy strain) [27] was derived from the first passage of the virus in Vero cells after rescue from the infectious clone. Briefly, CVB3 infectious clone [28] was linearized with SapI (New England Biolabs [NEB], Ipswich, MA, USA) and used to generate RNA in vitro (NEB). This RNA was transfected into Vero cells to recover the virus. The 2A^29K^ and 3C^52R^ mutant viruses were generated via site-directed mutagenesis of the wildtype CVB3 plasmid using the primers listed in Table 1 via mutagenic PCR with Phusion polymerase (Thermo-Fisher).

### 2.3. Infection and Enumeration of Viral Titers

CVB3 (Nancy strain) was derived from the first passage of virus in Vero cells (NR-10385), which were obtained through BEI Resources, National Institutes of Allergy and Infectious Diseases, National Institutes of Health (NR-10385). For all infections, DFMO and diethylnorsmperidine (DENSpm) were maintained throughout infection as designated. Viral stocks were maintained at −80 °C. For infection, the virus was diluted in serum-free DMEM for a multiplicity of infection (MOI) of 0.1 on Vero cells, unless otherwise indicated. The viral inoculum was overlain on cells for 10 to 30 min, and the cells were washed with PBS before replenishment of media. Supernatants were collected from CVB3 24 hpi and 48 hpi. Dilutions of cell supernatant were prepared in serum-free DMEM and used to inoculate confluent monolayer of Vero cells for 10 to 15 min at 37 °C. Cells were overlaid with 0.8% agarose in DMEM containing 2% NBCS. CVB3 samples incubated for 2 days at 37 °C. Following appropriate incubation, cells were fixed with 4% formalin and revealed with crystal violet solution (10% crystal violet; Sigma-Aldrich, St. Louis, MO, USA). Plaques were enumerated and used to back-calculate the number of plaque forming units (pfu) per milliliter of collected volume.

### 2.4. Mutant CVB3 Propagation

Vero cells were treated with DFMO for four days prior to infection with CVB3 at MOI 0.1. After 24 h, 1/10th of the cell culture volume was used to inoculate the next virus passage. This process was continued for ten passages, at which time RNA was purified from the cellular supernatant, reverse transcribed, amplified using CVB3-specific primers, and Sanger sequenced. Sequences were aligned to CVB3 parental genome and mutants were confirmed by manual chromatogram verification.

### 2.5. Drug Treatments

Difluoromethylornithine (DFMO; TargetMol, Boston, MA, USA) and *N*1,*N*11-Diethylnorspermine (DENSpm; Santa Cruz Biotechnology, Dallas, TX, USA) were diluted to 100× solution (100 mM and 10 mM, respectively) in sterile water. For DFMO treatments, cells were trypsinized and reseeded with fresh medium supplemented with 2% serum. Following overnight attachment, cells were treated with 100 µM, 500 µM, 1 mM, or 2 mM DFMO. Cells were incubated with DFMO for 96 h to allow for depletion of polyamines in Vero cells. For DENSpm treatment, cells were treated with 100 nM, 1 µM, 10 µM, 100 µM, and 1 mM 16 h prior to infection. During infection, media was cleared and saved from the cells. The same medium containing DFMO and DENSpm was then used to replenish the cells following infection. Cells were incubated at the appropriate temperature for the duration of the infection.

### 2.6. RNA Purification and cDNA Synthesis

Media was cleared from cells and Trizol reagent (Zymo Research, Irvine, CA, USA) directly added. The lysate was then collected, and RNA was purified according to the manufacturer’s protocol utilizing the Direct-zol RNA Miniprep Plus Kit (Zymo Research). Purified RNA was subsequently used for cDNA synthesis using High Capacity cDNA Reverse Transcription Kits (Thermo-Fischer), according to the manufacturer’s protocol, with 10–100 ng of RNA and random hexamer primers.

### 2.7. DFMO and DENSpm Sensitivity Assays

Vero cells were treated with either 100 µM to 2000 µM DFMO for 4 days or 1 µm to 100 µM DENSpm for 16 h prior to infection with CVB3 at an MOI of 0.1. At 24 hpi, supernatants were collected and titers were determined.

### 2.8. Stability and Fitness Assays

To measure the stability of the mutations, Vero cells were infected at an MOI of 0.1 with all of the viral mutants for 24 h. The virus was then passed to new cells by transferring 50 µL supernatant. After five passages, RNA was extracted and purified from supernatants and reverse transcribed. Sanger sequencing was used to determine whether mutations were stable over the passages by looking at the chromatograms and determining the presence or absence of the mutant nucleotide. Fitness assays were similarly performed, but Vero cells were infected at an MOI of 0.1 with an equal combination of wild-type and mutant CVB3 and passaged five times. Fitness was determined via Sanger sequencing and analysis of the chromatogram to determine if the wild-type or mutant nucleotide was present in the sample.

### 2.9. Protease Plasmid Cloning

Primers were designed to target either the wild-type 2A or 3C protease. CVB3 plasmids containing either the 2A mutant protease or 3C mutant protease were used to clone the mutant proteases. To target the 2A and 2A mutant proteases, the primers included SacI and XbaI recognition sites (Table 1). Primers against the 3C and 3C mutant proteases included NotI and XbaI recognition sites. Protease sequences were amplified via PCR and cloned into the pFLAG-CMV-LAP1 vector. Clones were verified for sequencing (GenScript, Piscataway, NJ, USA). Oligonucleotide sequences corresponding to the amino acid sequence for the wild-type and mutant 2A and 3C protease targets were designed and cloned into the pGlo-3F vector and verified by sequencing (GenScript).

### 2.10. Transfections

293T cells were plated at 80%–90% confluency and either treated with 100 µM, 500 µM, 1000 µM or 2000 µM DFMO for 4-days or left untreated. The plasmids were then transfected in the combinations described in the figures, according to the manufacturer’s protocol, using LipoD293 (SignaGen Laboratories, Rockville, MD, USA). The transfection was incubated at 37 °C for 24 h.

### 2.11. Luciferase Protease Assay

293T cells were treated with either 100 µM, 500 µM, 1000 µM, or 2000 µM DFMO for 4 days or left untreated. They were then transfected using LipoD293 (SignaGen Laboratories) with the 2A substrate alone, 3C substrate alone, 2A substrate plus the 2A WT protease or 2A mutant protease, or the 3C substrate plus the 3C WT protease or the 3C mutant protease and a TK Renilla transfection control plasmid. For luciferase assays, cells were combined with firefly substrate (Bright-Glo; Promega, Madison, WI, USA) followed by subsequent Renilla (Stop and Glo; Promega) luciferase substrate 24 h post-transfection. Luciferase assays were performed according to the manufacturer’s recommendations (Promega) and results were measured via the Veritas Microplate Luminometer (Turner BioSystems, Promega, Madison, WI, USA).

### 2.12. Western Blot

Samples were collected with Bolt LDS Buffer and Bolt Reducing Agent (Invitrogen, Waltham, MA, USA) and run on polyacrylamide gels. Gels were transferred using the iBlot 2 Gel Transfer Device (Invitrogen). Membranes were probed with primary antibodies for eIF4G, (1:1000, Santa Cruz Biotechnology, Dallas, TX, USA), GAPDH (1:1000, Santa Cruz Biotechnology), and β-actin (1:5000, Santa Cruz Biotechnology). Membranes were treated with SuperSignal West Pico PLUS Chemiluminescent Substrate (ThermoFisher Scientific) and visualized on FluorChem E imager (Protein Simple, San Jose, CA, USA).

### 2.13. Plaque Size Measurement

To quantify the relative plaque sizes of the different CVB3 mutants, Vero cells were seeded in 10 cm dishes and grown to confluence. Approximately 30 plaque-forming units (PFU) of each mutant was diluted in a 2.5 mL inoculum of serum-free DMEM. The media on the Vero cells was aspirated and replaced with the 2.5 mL inoculum containing the virus. The inoculum was incubated on the cells for approximately 30 min at 37 °C. After 30 min, an overlay of 8 mL 0.8% agarose was added to each dish. The dishes were incubated at 37 °C for 2 days to allow plaque formation. The cells were fixed with 4% formalin and the agarose plugs removed. The fixed cells were stained with crystal violet. Plaque size was determined using ImageJ software (Version 1.51k) [29].

### 2.14. Thin Layer Chromatography Determination of Polyamines

Polyamines were separated by thin-layer chromatography as previously described [30]. For all samples, cells were treated as described prior to being trypsinized and centrifuged. Pellets were washed with PBS and then resuspended in 200 µL 2% perchloric acid. Samples were then incubated overnight at 4 °C. 200 µL of supernatant was combined with 200 µL 5 mg/mL dansyl chloride (Sigma Aldrich) in acetone and 100 µL saturated sodium bicarbonate. Samples were incubated in the dark overnight at room temperature. Excess dansyl chloride was cleared by incubating the reaction with 100 µL 150 mg/mL proline (Sigma Aldrich). Dansylated polyamines were extracted with 50 µL toluene (Sigma Aldrich) and centrifuged. Five microliter of sample was added in small spots to the TLC plate (silica gel matrix; Sigma Aldrich) and exposed to ascending chromatography with 1:1 cyclohexane: ethyl acetate. The plate was dried and visualized via exposure to UV.

### 2.15. Statistical Analysis

Prism 6 (GraphPad) was used to generate graphs and perform statistical analysis. For all analyses, two-tailed Student’s *t*-test was used to compare groups, unless otherwise noted, with a = 0.05. For tests of sample proportions, *p* values were derived from calculated Z scores with two tails and a = 0.05. Half-maximal inhibitory concentration (IC50) values were calculated using Prism 6 using the built-in analysis tool.

## 3. Results

### 3.1. CVB3 Gains Resistance to Polyamine Depletion over Passages with DFMO

**Figure 1:**
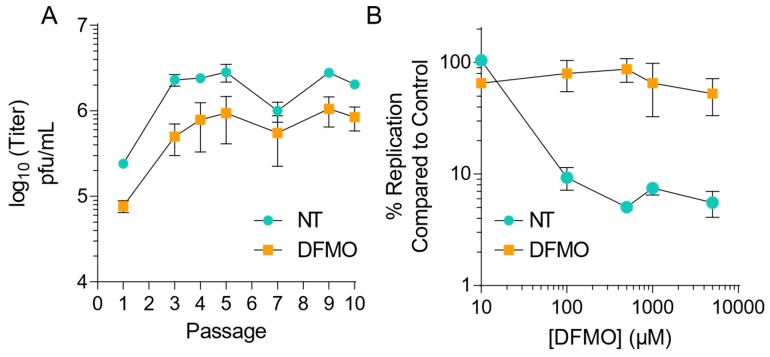
CVB3 gains resistance to polyamine depletion over passage with difluoromethylornithine. (**A**) Vero-E6 cells were left untreated or treated with 500 μM DFMO for four days prior to infection with CVB3 at MOI 0.1. Virus was collected at 24 hpi and used to inoculate the next passage. Viral titers were determined by plaque assay for the passages as shown. (**B**) CVB3 passaged five times over Vero-E6 cells, either treated with 500 μM DFMO or untreated, were used to infect Vero cells treated with increasing doses of DFMO for 24 hpi. Viral titers were determined by plaque assay. Error bars represent ± 1 SEM.

Our previous work demonstrating that CVB3 was sensitive to polyamine depletion via DFMO suggested a role for polyamines in virus replication [25]. To ascertain how CVB3 evolves in response to repeated replication cycles in the absence of polyamine depletion, we passaged the virus in the presence of DFMO. We treated Vero-E6 cells with 500 µM DFMO or left controls untreated (NT), for four days prior to infection with CVB3 at MOI 0.1 plaque forming units (pfu)/mL. After 24 h, we collected cellular supernatant and passaged 1/10th of the volume onto naïve cells. Viral titers were determined intermittently (Figure 1A) and at passage five, the passaged virus was exposed to increasing doses of DFMO, from 10 µM to 5 mM, and titers measured by plaque assay after 24 h of infection. We observed that virus that had been passaged but not exposed to DFMO remained susceptible, with significant decreases in viral titer observed at DFMO concentrations above 100 µM and an IC50 value of 70.9 µM. In contrast, virus passaged in DFMO exhibited significant resistance to DFMO treatment, with an IC50 value of 293 µM, suggesting developing resistance to DFMO.

Following passage of virus, RNA was extracted, purified, reverse transcribed and Sanger sequenced. When the sequenced virus was aligned with the parental CVB3 genome, mutations in the two viral proteases, 2A (Q29K) and 3C (Q52R), were observed. Interestingly, these mutations were observed independently in three distinct viral lineages. Further, the 2A^29K^ and 3C^52R^ mutations were not observed in the same lineage. Importantly, no mutations were observed in viruses passaged without treatment.

### 3.2. CVB3 Mutations in 2A and 3C Proteases Confer Resistance to Polyamine Depletion

**Figure 2:**
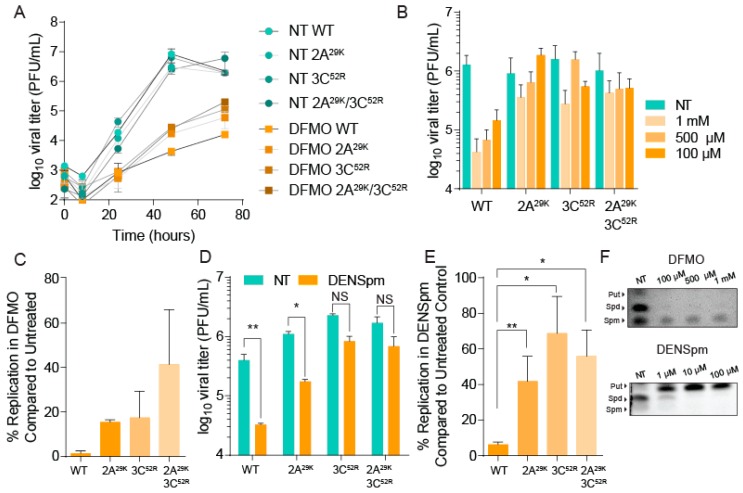
CVB3 mutations in 2A and 3C proteases confer resistance to polyamine depletion. (**A**) Vero cells were left untreated or treated with 500 μM DFMO for four days prior to infection with CVB3 and 2A and 3C protease mutants. Samples were collected every 24 h and titered via plaque assay. (**B**) Vero cells were treated with increasing doses of DFMO, from 100 μM to 1 mM, for four days prior to infection with wildtype CVB3 or protease mutants. Viral titers were determined by plaque assay at 48 hpi. (**C**) Viral titers from (**A**) were used to calculate the percent replication in DFMO, by dividing the titer of the virus after infection of DFMO-treated cells by the titer of the virus after infection of untreated cells at 48 hpi. (**D**) Vero cells were left untreated or treated with 100 μM DENSpm for 16 h prior to infection with wildtype and mutant CVB3 at MOI 0.1. Viral titers were determined by plaque assay at 24 hpi. (**E**) Percent replication was determined as in (**C**) but using titers from (**D**). (**F**) Thin-layer chromatograms resolving the polyamines putrescine (Put), spermidine (Spd) and spermine (Spm) after treatment with DFMO (above) and DENSpm (below). * *p* ≤ 0.05, ** *p* ≤ 0.01, *** *p* ≤ 0.001 using Student’s *t*-test (*n* ≥ 3), comparing treated samples to untreated controls. Error bars represent ± 1 SEM.

Observing the development of resistance to DFMO and the emergence of mutants in the passaged virus, we cloned these mutations in the CVB3 parental strain using mutagenic PCR on the CVB3 infectious clone. In addition to 2A^29K^ and 3C^52R^ mutants, we generated a 2A^29K^/3C^52R^ double mutant. After confirming the presence of the mutations, we generated an infectious virus and measured the virus titer over several rounds of replication. Vero cells were treated with 500 µM DFMO or left untreated for four days prior to infection at MOI 0.01. Cellular supernatant samples were collected and titered via plaque assay. As shown in Figure 2A, all viruses in untreated conditions replicated with similar kinetics and to similar levels regardless of protease mutation. However, in DFMO-treated cells, the wildtype CVB3 exhibited significant reductions in titer, which persisted to 72 hpi. Interestingly, the protease mutants, either alone or in combination, exhibited a significant rescue in viral titer compared to the wildtype virus, suggesting partial resistance to polyamine depletion.

To quantify resistance, we measured viral titers of wildtype and protease-mutant virus in untreated and DFMO-treated Vero cells over a concentration range (Figure 2B). We infected these cells at MOI 0.1 and measured viral titers at 48 hpi. We again observed significant sensitivity by the wildtype virus with any concentration of DFMO; in contrast, both protease mutants exhibited significant resistance, again suggesting that these mutations confer resistance to polyamine depletion. We then measured percent replication by dividing the titer in DFMO treatment conditions (500 µM) by the titer in untreated conditions from 48 hpi (Figure 2C). We observed that compared to wildtype virus, all mutants exhibited higher replication, and the protease mutations conferred similar levels of resistance. Combination of the mutations appeared to additively enhance replication; however, this difference was not statistically significant.

While these mutations were obtained from a serial passage in DFMO-treated cells, we wished to determine whether these mutations conferred resistance to polyamine depletion and were not a consequence of a pleiotropic effect from DFMO. Toward this end, we treated cells with a different polyamine-depleting agent, diethylnorspermidine (DENSpm), which acts to accelerate polyamine catabolism by acetylating spermidine and spermine. Vero cells were treated with 100 µM DENSpm for 16 h prior to infection at MOI 0.1 with wildtype and mutant CVB3. At 48 hpi, we measured viral titers, and again we observed significant sensitivity with the wildtype virus (Figure 2D). However, we observed partial resistance in our protease mutant viruses. As with DFMO, when we calculated the percent replication in DENSpm-treated cells compared to untreated cells, the protease mutants exhibited resistance that was additive (Figure 2E). We verified that both of these molecules were depleting polyamines in our treated cells by directly visualizing polyamines via thin-layer chromatography (Figure 2F).

### 3.3. CVB3 Stability, Fitness, and Specific Infectivity Are Not Altered with Protease Mutation

**Figure 3:**
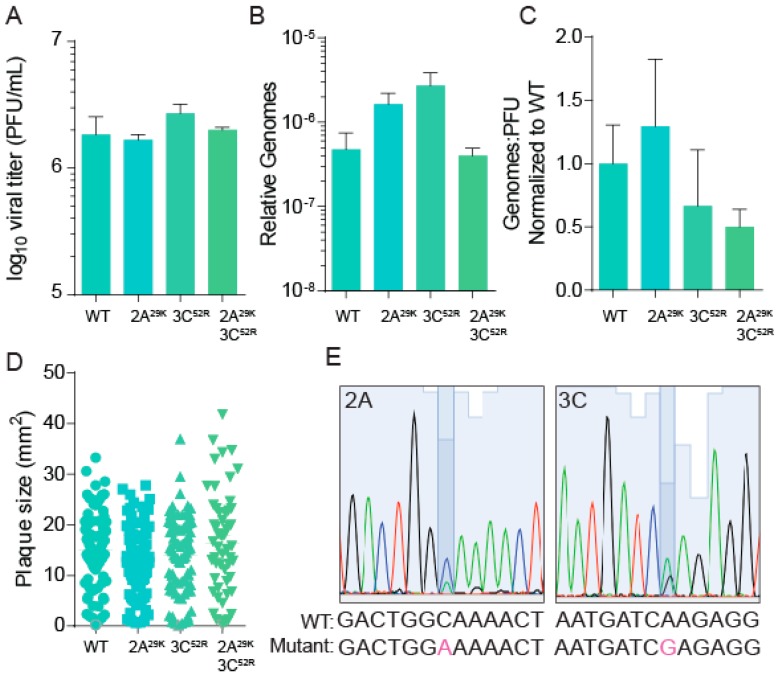
CVB3 stability, fitness, and specific infectivity are not altered with protease mutation. Vero cells were infected with wildtype and mutant CVB3 for 24 h, at which time (**A**) viral titers were determined by plaque assay and (**B**) viral genomes in cell supernatant were determined by qPCR. (**C**) The ratio of genomes-to-PFU was calculated by dividing the relative genomes in (**B**) by the titer in (**A**). (**D**) Plaque sizes of virus mutants were determined after a two-day plaque assay. (**E**) WT and mutant CVB3 were used to infect untreated Vero cells for five passages at which time viral RNA was reverse transcribed, PCR amplified and sequenced. Highlighted nucleotide indicates mutant.

To determine whether our protease-mutant viruses differed significantly from our wildtype virus, we characterized fitness and replication characteristics. As shown in Figure 2A, the wildtype and mutant viruses replicate with similar kinetics. To ascertain whether these viruses produced similar levels of infectious particles, we measured the ratio of viral genomes to infectious virus. Vero cells were infected with the wildtype or protease mutant viruses for 48 h, and subsequently, viral titers (Figure 3A) were determined by plaque assay and RNA was processed from infected cellular supernatant. The number of viral genomes was quantified by qRT-PCR (Figure 3B), and the ratio of genomes to infectious virus (pfu) calculated (Figure 3C). We observed no changes in viral titers in polyamine-sufficient conditions, and viral genomes were slightly elevated, though not statistically significant, in 2A^29K^ and 3C^52R^ mutant viruses (Figure 3B). Overall, the genome-to-pfu ratio was not statistically significantly different among our protease mutant viruses.

Passaging CVB3 in Vero cells can result in the emergence of a virus with fitness changes that provide a replicative advantage, as we had observed with our CHIKV mutant viruses [31]. To determine whether our CVB3 mutants had fitness enhancement manifesting as a more rapidly replicating virus, we measured virus plaque sizes of wildtype and protease mutant virus. We observed no significant difference between wildtype and mutant CVB3 plaque size (Figure 3D). To better measure viral fitness via competition assay, we infected Vero cells with equal quantities of infectious virus of wildtype and 2A^29K^ or 3C^52R^, and after five passages, we extracted RNA, reverse transcribed, amplified viral genomes by PCR, and Sanger sequenced the appropriate genomic locus corresponding to each protease (Table 1). After the fifth passage, we observed that the wildtype and either protease mutant virus existed as a mixed population, with peaks corresponding to the wildtype and mutant nucleotide present in the samples (Figure 3E, representative of three independent passages). Additionally, we passaged mutant virus without competition to determine the stability of the mutant. We observed that the mutation was maintained after five passages, suggesting that there is no fitness cost to this mutation in our cell culture conditions. Altogether, our data suggest that protease-mutant CVB3 exhibits no significant replicative advantage compared to wildtype CVB3.

### 3.4. CVB3 Protease Activity Is Modulated by Cellular Polyamine Levels

**Figure 4:**
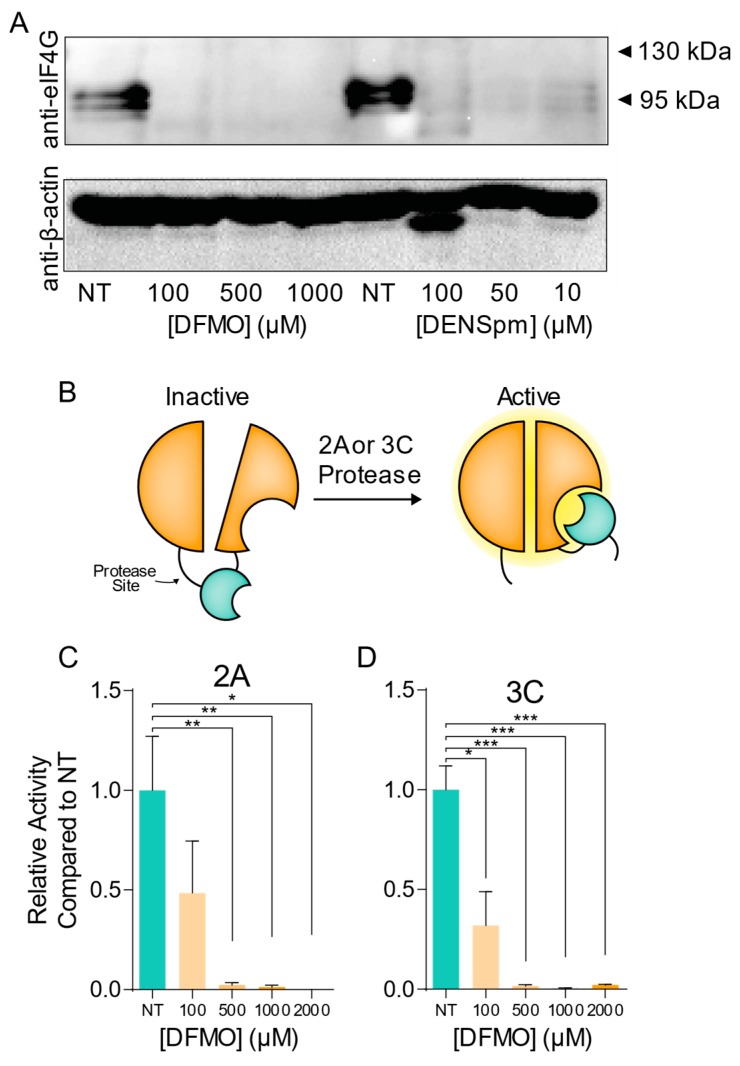
CVB3 protease activity is modulated by cellular polyamine levels. (**A**) Veros were left untreated or treated with increasing doses of DFMO for four days or increasing doses of DENSpm for 16 h prior to infection with wildtype CVB3. Total cellular protein was collected at 24 hpi and analyzed via western blot for eIF4G and β-actin. (**B**) Split luciferase protease activity reporter systems were cloned as shown and co-transfected with (**C**) 2A and (**D**) 3C into 293T cells left untreated or treated with increasing doses of DFMO. Firefly luciferase activity was measured 24 h later and normalized to renilla luciferase transfection efficiency control and subsequently normalized to untreated cell transfection. * *p* ≤ 0.05, ** *p* ≤ 0.01, *** *p* ≤ 0.001 using Student’s *t* test (*n* ≥ 3), comparing treated samples to untreated controls. Error bars represent ± 1 SEM.

Given that we had observed resistance mutations in both 2A and 3C proteases after passaging virus in polyamine-depleted cells, we hypothesized that viral protease activity may be modulated by cellular polyamines. While previous reports have suggested that polyamines enhance chymotrypsin activity [32,33], a role for polyamines in viral protease activity has never been reported. Toward this end, we treated cells with increasing doses of DFMO or DENSpm to deplete cellular polyamines. Following incubation with a drug, cells were infected at MOI 10 for 24 h, at which time cellular lysates were collected for western analysis. Lysates were analyzed for the presence of cleaved eIF4G, a cellular target of the viral proteases. We observed that in infected cells, a distinct eIF4G band was present; however, in cells treated either with DFMO or DENSpm, eIF4G cleavage was significantly restricted (Figure 4A). We were unable to visualize full-length eIF4G, likely due to its large size.

The reduction in eIF4G cleavage by viral protease in the presence of polyamine depleting molecules could be the result of polyamines modulating viral protease activity, or the phenotype could be due to restriction of virus replication, protease translation, and subsequent reduction in proteolytic activity. To directly measure viral protease activity in cells, we generated a protease-sensitive dual luciferase reporter system. In this split luciferase system, cleavage of a target sequence allows for robust firefly luciferase activity, which can be normalized to a renilla luciferase transfection control. This system has previously been used to explore the activity of viral polymerases [34,35,36], though it has not been considered in the context of polyamine depletion. To generate this system, we cloned a 2A target sequence—MTNTGAFG—and a 3C target sequence—AMEQG—into the firefly luciferase reporter [37]. We also cloned 2A and 3C into the pCMV plasmid to drive robust protease expression. These constructs were cotransfected into 293T cells either left untreated or treated with increasing doses of DFMO. Firefly activity was measured 24 h later and normalized to renilla activity. We observed that for both 2A (Figure 4C) and 3C (Figure 4D), protease activity was significantly reduced with DFMO treatment, to levels that were nearly undetectable with DFMO concentrations at or above 500 µM. These data support our hypothesis that polyamines modulate viral protease activity, and polyamine depletion restricts virus replication by quelling viral protease activity.

### 3.5. CVB3 Protease Mutations Enhance Proteolytic Activity in Polyamine-Depleted Cells

**Figure 5:**
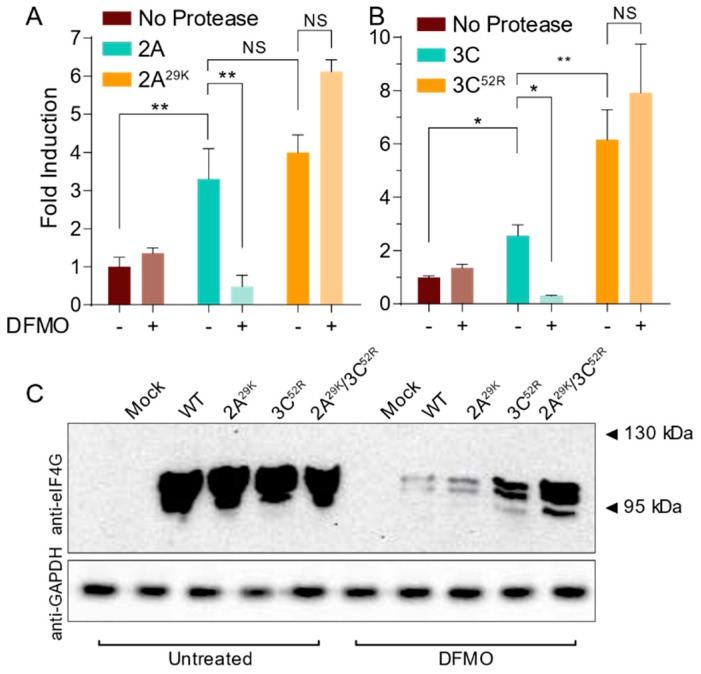
CVB3 protease mutations enhance proteolytic activity in polyamine-depleted cells. (**A**) 293T cells were treated with 500 μM DFMO or left untreated and transfected with empty vector, wildtype 2A, and mutant 2A. Protease activity was measured via dual luciferase assay at 24 h. (**B**) 293T cells were treated and transfected as in (A) but using wildtype and mutant 3C constructs. (**C**) Vero cells were left untreated or treated with 500 μM DFMO for four days prior to infection with wildtype and protease mutant CVB3. At 24 hpi, total cellular protein was collected and analyzed for eIF4G and GAPDH. * *p* ≤ 0.05, ** *p* ≤ 0.01, *** *p* ≤ 0.001, or not significant (NS) using Student’s *t* test (*n* ≥ 3), comparing treated samples to untreated controls. Error bars represent ± 1 SEM.

With resistance mutations emerging in CVB3 2A and 3C proteases, we next hypothesized that these mutations may enhance viral replication in polyamine-depleted cells by maintaining proteolytic activity. To test this, we generated mutant 2A^29K^ and 3C^52R^ proteases through site-directed mutagenesis of our overexpression vectors. We then cotransfected these proteases and the wildtype controls with our luciferase reporter system into untreated and 500 µM DFMO-treated Veros. At 24 h, we measured luciferase activity and normalized to renilla luciferase activity and to control cells transfected with luciferase reporter alone (no protease). We observed that both 2A^WT^ and 3C^WT^ exhibited protease activity that was DFMO-sensitive (Figure 5A,B). Interestingly, we also observed that both 2A^29K^ and 3C^52R^ exhibited robust protease activity, even in DFMO-treated cells. We also observed that 3C^52R^ exhibited higher protease activity than 3C^WT^, though this was not observed for 2A.

We further hypothesized that mutant proteases may enhance proteolytic cleavage of a cellular target during viral infection to enhance virus replication. To this end, we treated Veros with 500 µM DFMO or left cells untreated and then infected at MOI 10 for 24 h. Cellular lysates were collected and analyzed for cleavage of eIF4G (Figure 4C). In untreated cells, we observed significant cleavage of eIF4G with wildtype infection that was not enhanced with infection of CVB3-2A^29K^, -3C^52R^, or double protease mutant. However, with DFMO treatment, eIF4G cleavage was significantly reduced in wildtype CVB3 infection, as previously observed (Figure 4A). When we analyzed DFMO-treated cells that were infected with protease mutant CVB3, we observed proteolytic cleavage of eIF4G, significantly higher than wildtype CVB3. In total, our data suggest that polyamine depletion significantly reduces CVB3 protease activity and that the mutant proteases derived from our passages exhibit a rescue in this sensitivity.

## 4. Discussion

Polyamines play several roles in the eukaryotic cell and likely affect virus replication at several stages. By isolating DFMO escape mutants, we can glean insight into the mechanisms by which polyamines function in virus infection. Here, we have isolated two protease mutants following passage of CVB3 in DFMO. We previously characterized escape mutants for chikungunya virus and found three mutations that conferred resistance when simultaneously present in the virus [31]. In contrast, we find that CVB3 gains resistance to DFMO with a single mutation in either protease. From our data, we cannot determine whether polyamines facilitate protease activity against a specific target; however, we observe that both viral proteases maintain activity in polyamine-depleted cells with either of these mutations and that these mutations facilitate virus infection in an otherwise antiviral environment. Determining the specificity of this activity is an area of important future investigation.

In the course of passaging CVB3 in the presence of DFMO, we observed two mutations changing from glutamine to positively charged lysine or arginine. Interestingly, we have observed such amino acid changes in our CHIKV passages [31], in which we observed a glycine-to-arginine mutation in nsP1. Given that polyamines are positively-charged at physiological pH, the emergence of positively-charged amino acids in polyamine-depleted conditions suggests potential amino acid compensation for a loss of charge. The amino acids in which we observe mutations for CVB3 2A and 3C are not located in the active sites of either enzyme [27,38], suggesting that these amino acids and their associated charge may alter enzymatic activity allosterically. Precisely how positively-charged amino acids or polyamines contribute to protease activity remains unknown.

Others have observed that polyamines contribute to protease activity [39,40]. Prior research in oat leaves identified polyamines as factors that reduce cellular protease activity and, as a result, leaf senescence [41]. The function of polyamines in plant tissues appears distinct from the functions in mammalian eukaryotic cells, though. For instance, spermidine interacts with and activates the activity of mammalian chymotrypsin [32,33]. Whether polyamines function broadly in the function of proteases, especially viral proteases, has not been fully explored; however, our work suggests that viral proteases may be modulated by polyamines.

Targeting polyamines as an antiviral strategy shows significant promise, and the availability of FDA-approved molecules like DFMO underscores the potential of inhibiting this pathway for limiting viral infection. We previously described how DFMO reduces CVB3 replication in vitro and in vivo [25]. Here, we show that CVB3 gains resistance to DFMO-mediated polyamine depletion via two distinct and independent mutations. Such emergence of resistance highlights that polyamine depletion as a monotherapy may not be sufficient to quell virus replication. As viruses rapidly mutate and gain resistance to antivirals, potential therapy that includes polyamine depletion would necessitate combination therapy to prevent resistance. Neither protease mutant exhibited an enhanced fitness phenotype, as we had observed with CHIKV mutants [31], suggesting that even if this virus were to emerge, the mutation may not fix in a viral population not exposed to DFMO. 

Beyond underscoring combination therapy, viral escape mutants are powerful tools for understanding how antivirals function during viral infection. By investigating how CVB3 escaped DFMO-mediated polyamine depletion, we observed novel mutations in viral proteases that led us to investigate how polyamines contribute to viral protease activity. Thus, these escape mutants have highlighted a novel function of polyamines during viral infection. As described above, these results have implications for antiviral therapy, but they also inform our understanding of basic mechanisms in virology. Additional mechanisms whereby polyamines function in virus infection may be observed using similar means to highlight how these relatively simple molecules contribute to infection in diverse ways.

## Figures and Tables

**Table 1:** Primers used in this study.

Protease/Target	Function	Sense	Sequence
2A	Clone	F	5’-GCTGCGGCCGCAGGCGCATTTGGACAACAATCAGG-3’
	Clone	R	5’-GCTTCTAGATTACTGTTCCATTGCATCATCCTTCCAG-3’
	Sequence	F	5’-CATGTCAAAGCGTGGATACCTAGAC-3’
	Sequence	R	5’-GCACATGGGATTGGTATCTCCTGGG-3’
	qPCR	F	5’-CATGTCAAAGCGTGGATACCTAGAC-3’
	qPCR	R	5’-GCACATGGGATTGGTATCTCCTGGG-3’
3C	Clone	F	5’-GTTGCGGCCGCTGGCCCTGCCTTTGAGTTCGCCG-3’
	Clone	R	5’-GCGTCTAGATTATTGCTCATCATTGAAGTAGTGTTTG-3’
	Sequence	F	5’-TATACAGGAGTGCCCAACCAGAAGC-3’
	Sequence	R	5’-GAATGTACATGTTGGGAAACTTGCT-3’
	qPCR	F	5’-AGGGCGAGATCAATCACATTAG-3’
	qPCR	R	5’-CTCTGCTGTTGCCTCACTATC-3’
2A ProTarget	Clone	F	5’-GATCCATGACCAACACCGGCGCGTTTGGCTAA-3’
	Clone	R	5’-AGCTTTAGCCAAACGCGCCGGTGTTGGTCATG-3’
3C ProTarget	Clone	F	5’-GATCCGCGATGGAACAGGGCTAA-3’
	Clone	R	5’-AGCTTTAGCCCTGTTCCATCGCG-3’
2A/3C ProTarget	Sequence	F	5’-GTAGTACAGACTGGAAAATATC-3’
